# Germ cell-intrinsic requirement for the homeodomain transcription factor PKnox1/Prep1 in adult spermatogenesis

**DOI:** 10.1371/journal.pone.0190702

**Published:** 2018-01-02

**Authors:** Yasuhiro Kawai, Akihisa Oda, Yoshiakira Kanai, Ryo Goitsuka

**Affiliations:** 1 Division of Development and Aging, Research Institute for Biomedical Sciences, Tokyo University of Science, Noda, Chiba, Japan; 2 Department of Veterinary Anatomy, The University of Tokyo, Bunkyo-ku, Tokyo, Japan; 3 Center for Animal Disease Models, Research Institute for Science & Technology, Tokyo University of Science, Noda, Chiba, Japan; National Cancer Institute, UNITED STATES

## Abstract

PKnox1 (also known as Prep1) belongs to the TALE family of homeodomain transcription factors that are critical for regulating growth and differentiation during embryonic and postnatal development in vertebrates. We demonstrate here that PKnox1 is required for adult spermatogenesis in a germ cell-intrinsic manner. Tamoxifen-mediated PKnox1 loss in the adult testes, as well as its germ cell-specific ablation, causes testis hypotrophy with germ cell apoptosis and, as a consequence, compromised spermatogenesis. In PKnox1-deficient testes, spermatogenesis was arrested at the c-Kit^+^ spermatogonia stage, with a complete loss of the meiotic spermatocytes, and was accompanied by compromised differentiation of the c-Kit^+^ spermatogonia. Taken together, these results indicate that PKnox1 is a critical regulator of maintenance and subsequent differentiation of the c-Kit^+^ stage of spermatogonia in the adult testes.

## Introduction

Spermatogenesis is a complex and highly ordered cell differentiation process in which the germ cell lineage gives rise to functional gametes in the male. During adult spermatogenesis in mice, spermatogonia are localized closely attached onto the basement membrane of seminiferous tubules, and their descendants are arranged towards the lumen. Distinct spermatogonia differentiation stages have been defined based on morphological features: A_single_ (A_s_; isolated single cells), A_paired_ (A_pr_; chains of 2 cells), and A_aligned_ (A_al_; chains of 4 or 8 cells) are referred to as early undifferentiated spermatogonia [1,2]. Subsequently, A_al_ cells give rise to the late undifferentiated spermatogonia (A_al16~32_), and then to differentiating spermatogonia (A1 to A4), which are committed to meiosis[3,4].

The balance between maintenance of the undifferentiated state and differentiation is controlled by a complex interplay of germ cell-intrinsic mechanisms and -extrinsic factors secreted by Sertoli cells that support germ cells within the seminiferous tubules[5]. Several transcription factors expressed in the germ cells have been implicated in the regulation of spermatogenesis, including PLZF[6,7], Taf4b[8] and SOHLH1/2[9,10]. With regard to Sertoli cell-derived factors, glial cell line-derived neurotrophic factor (GDNF) supports self-renewal of undifferentiated spermatogonia through binding to its receptor consisting of GFRα1and RET[11], while signaling from c-Kit, when bound by its ligand stem cell factor expressed by Sertoli cells, plays crucial roles in regulating proliferation, survival and the entry of spermatogonia into meiosis [12]. Furthermore, retinoic acid, the biologically active form of vitamin A supplied by Sertoli cells, has also been shown to regulate spermatogonia differentiation, as vitamin A-deficient mice are infertile because of an arrest of spermatogonia differentiation at the A_al_-A1 transition[13].

The three-amino-acid-loop-extension (TALE) class of homeodomain transcription factors are recognized as critical for regulating growth and differentiation during embryonic and postnatal development in vertebrates[14]. The TALE homeodomain transcription factors, including the Meis, PKnox and Pbx families, share a conserved atypical homeodomain through which they can bind to the target DNA as well as interact with Hox proteins[15]. In addition, PKnox and Meis family members have conserved protein interaction domains, MEIS-A and MEIS-B (also termed HM1 and HM2), in their N-terminal region that function as an interface for heterodimerization with Pbx family members, promoting their nuclear translocation and also affecting DNA-binding specificity[16–19]. PKnox1 (Pbx/Knotted homeobox 1), also known as Prep1, is expressed ubiquitously in embryonic and adult tissues but at distinct levels in different organs[20]. A PKnox1/Prep1 null mutation causes lethality shortly after implantation[21], while *PKnox1/Prep1*-hypomorphic mice display a leaky lethal phenotype characterized mostly by hematopoietic and angiogenic defects by embryonic day (E) 17.5[22]. In the adult mouse testes, another TALE family member, Pbx4, is preferentially expressed in the germ cell lineage[23], and has been reported to cooperate with PKnox/Meis family members to initiate testis-specific transcription of the *Pgk2* gene[24,25], suggestive of a potential involvement of PKnox1 in adult spermatogenesis. While the testis is one of the tissues where PKnox1 is highly expressed[20], defects in spermatogenesis have not been reported in the *PKnox1/Prep1*-hypomorphic mice.

In the present study, we thus examined the function of PKnox1 in adult spermatogenesis by generating PKnox1 conditional knockout mice and demonstrated that PKnox1 is required for the differentiation of the c-Kit^+^ stage of spermatogonia in a cell-intrinsic manner.

## Results

### PKnox1 is expressed in germ cells of the adult testes

**Fig 1:**
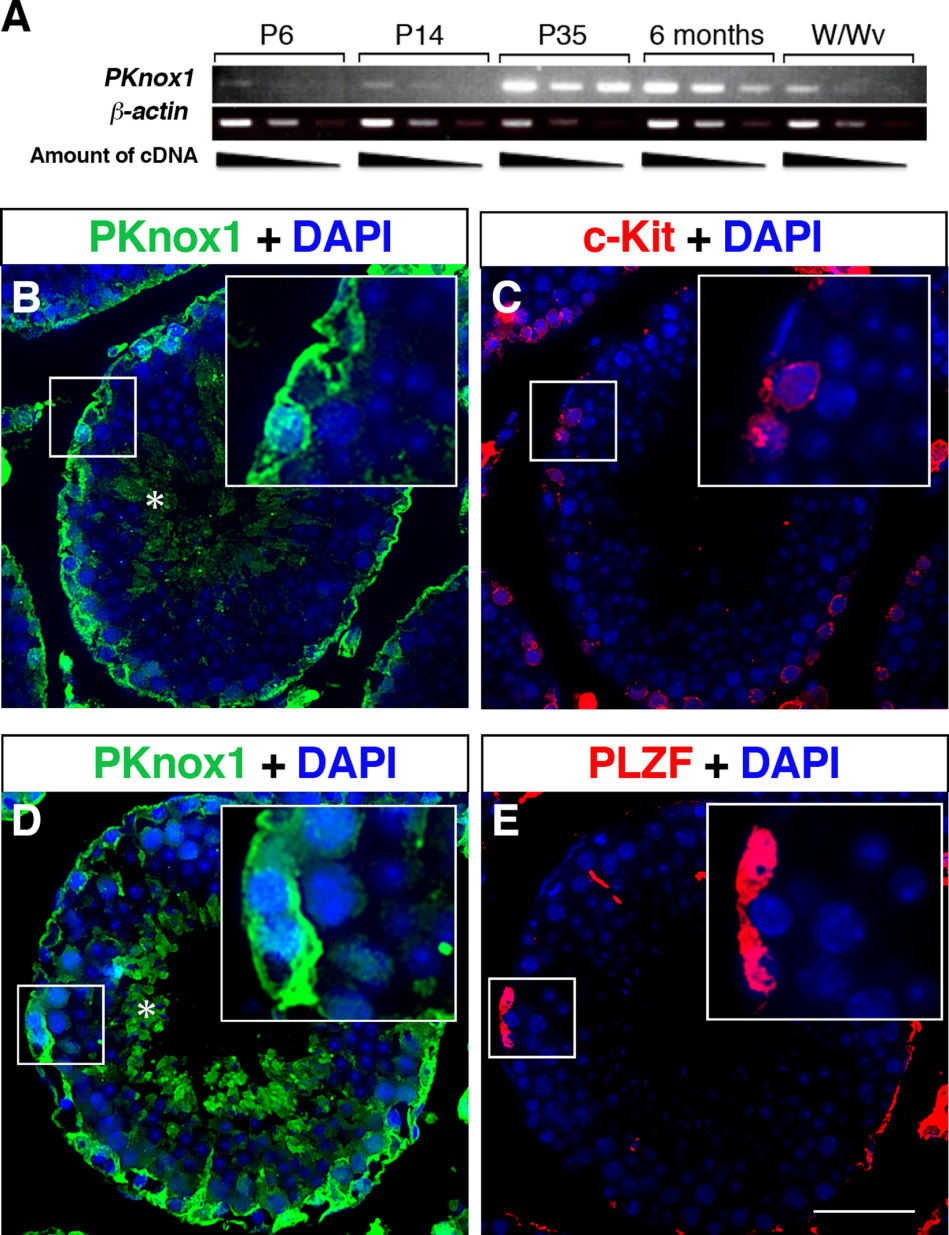
Expression of PKnox1 in postnatal testes. (**A**) Expression of *PKnox1* and *β-actin* (control) mRNA transcripts in testes at postnatal (P) days 6, 14, 35 and 6 months obtained from wild-type mice and 12-week-old *W/Wv* mice. (**B-E**) Immunohistochemical analysis to localize PKnox1-expressing cells in the adult testis. Serial tissue sections of the testes from 8-week-old wild-type mice were stained with a PKnox1-specific antibody, in combination with anti-c-Kit (**B**, **C**) or PLZF antibodies (**D**, **E**). Inserts indicate cells co-expressing both Prep1 and c-Kit or PLZF. Asterisks indicate haploid cells positive for PKnox1. Data are representative of 3 independent experiments. Scale bar, 50 μm.

We examined the expression of *PKnox1* in the testes at postnatal day (P) 6, 14, 35 and adult (6 months) by RT-PCR. *PKnox1* expression was first detectable at P6, when gonocytes have been shown to complete their migration to the basement membrane of the testes[5]. The expression level of Prep1 then increased with age and reached a plateau at P35, a time point of termination of the first wave of spermatogenesis (Fig 1A). In contrast to wild-type adult testes, only weak *PKnox1* expression was detected in the adult testes of *W/W*_*v*_ mice, which have an arrest in spermatogenesis at the c-Kit^+^ spermatogonia stage[26,27]. Immunohistochemical analysis revealed that a high level expression of PKnox1 was detected in most of the germ cells lining the basement membrane (Fig 1B and 1D), and some of it co-localized with cells expressing the late and early stage spermatogonia markers, c-Kit (insert in Fig 1B and 1C) and PLZF (insert in Fig 1D and 1E), respectively. In addition, expression of PKnox1 was also observed in haploid cells near the lumen of the seminiferous tubule (asterisks in Fig 1B and 1E). These findings suggest that PKnox1 is expressed in germ cells, from the early to the late stages of spermatogonia as well as at the haploid developmental stages in the adult testes.

### Loss of PKnox1 causes defects in adult spermatogenesis

As the early embryonic lethality resulting from germline deletion of the *PKnox1* gene precludes any study of spermatogenesis in the adult testes, we generated mice harboring conditional alleles of *PKnox1*, in which exon 3 of the *PKnox1* gene encoding the N-terminal part of the Pbx-binding domain is floxed by *lox*P sites (S1A Fig). Removal of exon 3 is predicted to induce a frameshift mutation, leading to the incorporation of 11 unrelated amino acids before encountering a termination codon within exon 4. Even if a truncated polypeptide generated from this mutant transcript could exist as a stable protein, the mutant protein would lack almost all functional PKnox1 domains, including the two Pbx-binding domains and the homeodomain, thus rendering *PKnox1* a null allele. To examine a potential involvement of PKnox1 in adult spermatogenesis, we chose to study the consequence of *PKnox1* ablation in the testes by crossing the mice carrying the *PKnox1*^*fl*^ allele with the *Rosa26* gene-driven tamoxifen-responsive *Rosa26-CreER*^*T2*^ knock-in mouse line, which causes highly efficient excision of *lox*P-flanked DNA in a ubiquitous manner after induction by tamoxifen[28]. Three intragastric administrations of tamoxifen into *Rosa26-CreER*^*T2*^*; PKnox1*^*fl/fl*^ mice were sufficient to induce efficient deletion of the floxed *PKnox1* locus and the loss of PKnox1 protein in the adult testes (S1B and S1C Fig).

**Fig 2:**
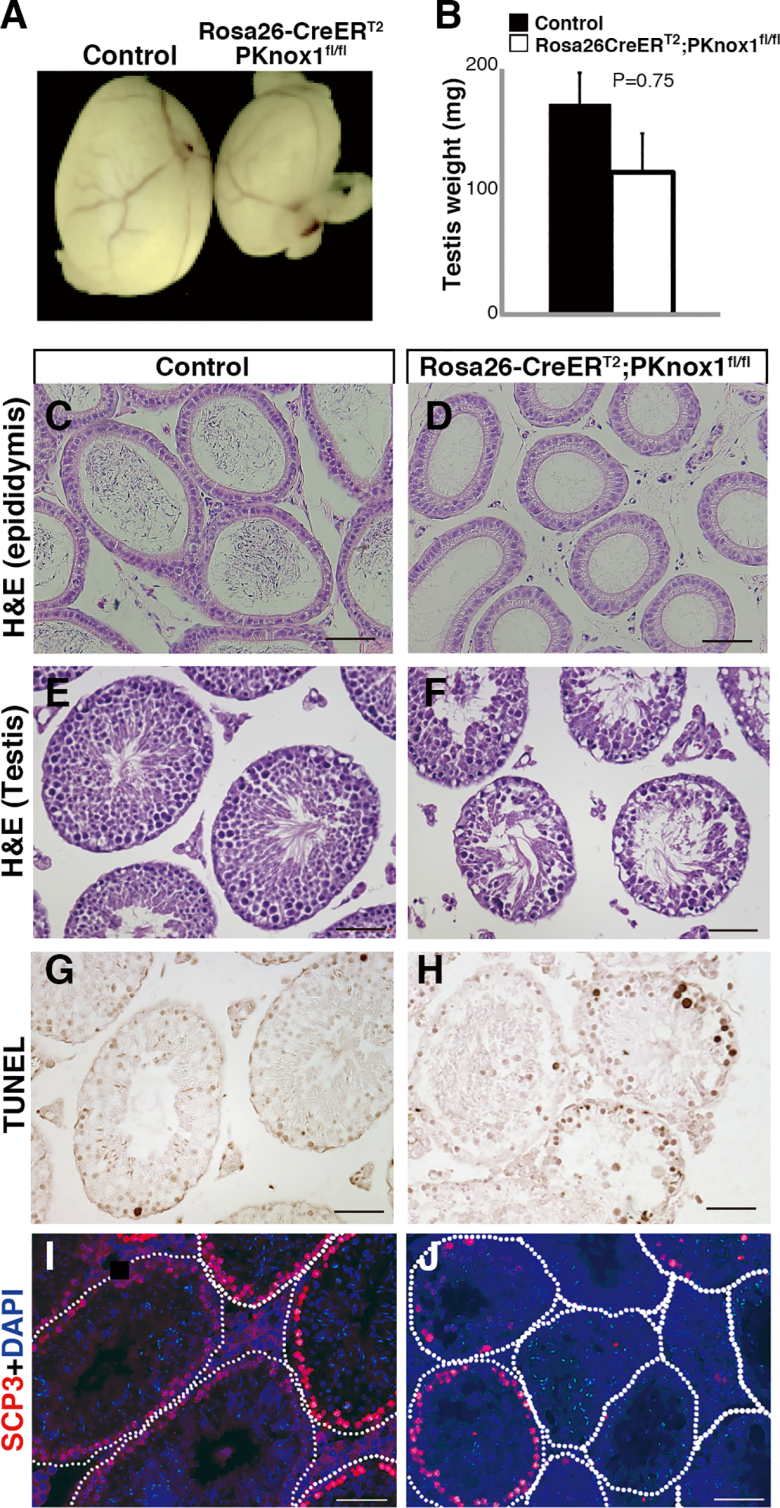
Loss of PKnox1 in the adult testis causes defective spermatogenesis. The size (**A**) and weight (**B**) of adult testes from 12-week-old PKnox1-CKO and control mice 3 weeks after induction of *PKnox1* deletion. Bars are the mean and standard deviation of the weight of testes. (n = 4 for each genotype). Tissue sections from control and CKO epididymis stained with H&E (**C**, **D**) and testes stained with H&E (**E, F**), TUNEL (**G, H**) and anti-SCP3 antibody with DAPI (**I, J**). Data are representative of 4 independent experiments. Scale bars, 50 μm.

We first analyzed spermatogenesis in adult *Rosa26-CreER*^*T2*^*; PKnox1*^*fl/fl*^ mice three weeks after tamoxifen treatment (hereafter referred to as PKnox1-CKO) and compared it to that of similarly treated *Rosa26-CreER*^*T2*^*; PKnox1*^*fl/+*^ control littermates. Three weeks after the induction of *PKnox1* deletion, the size and weight of the testis was markedly, albeit not significantly, reduced in PKnox1-CKO mice compared to similarly treated controls (Fig 2A and 2B). Histological analysis of epididymides from PKnox1-CKO mice did not contain sperms (Fig 2C and 2D), and examination of sections of testes revealed atrophied seminiferous tubules containing very few spermatocytes (Fig 2E and 2F), while germ cells beneath the basement membrane, probably representing spermatogonia and haploid cells, appeared to exist (Fig 2F). Furthermore, we observed a profound increase in TUNEL^+^ apoptotic cells in PKnox1-CKO testes when compared with those in controls (Fig 2G and 2H). Immunohistochemical analyses further revealed that most seminiferous tubules in PKnox1-CKO testes lack SCP3^+^ meiotic cells (Fig 2I and 2J). These findings indicate that PKnox1 is indispensable for spermatogenesis in the adult testes.

### PKnox1 regulates adult spermatogenesis in a germ cell-intrinsic manner

**Fig 3:**
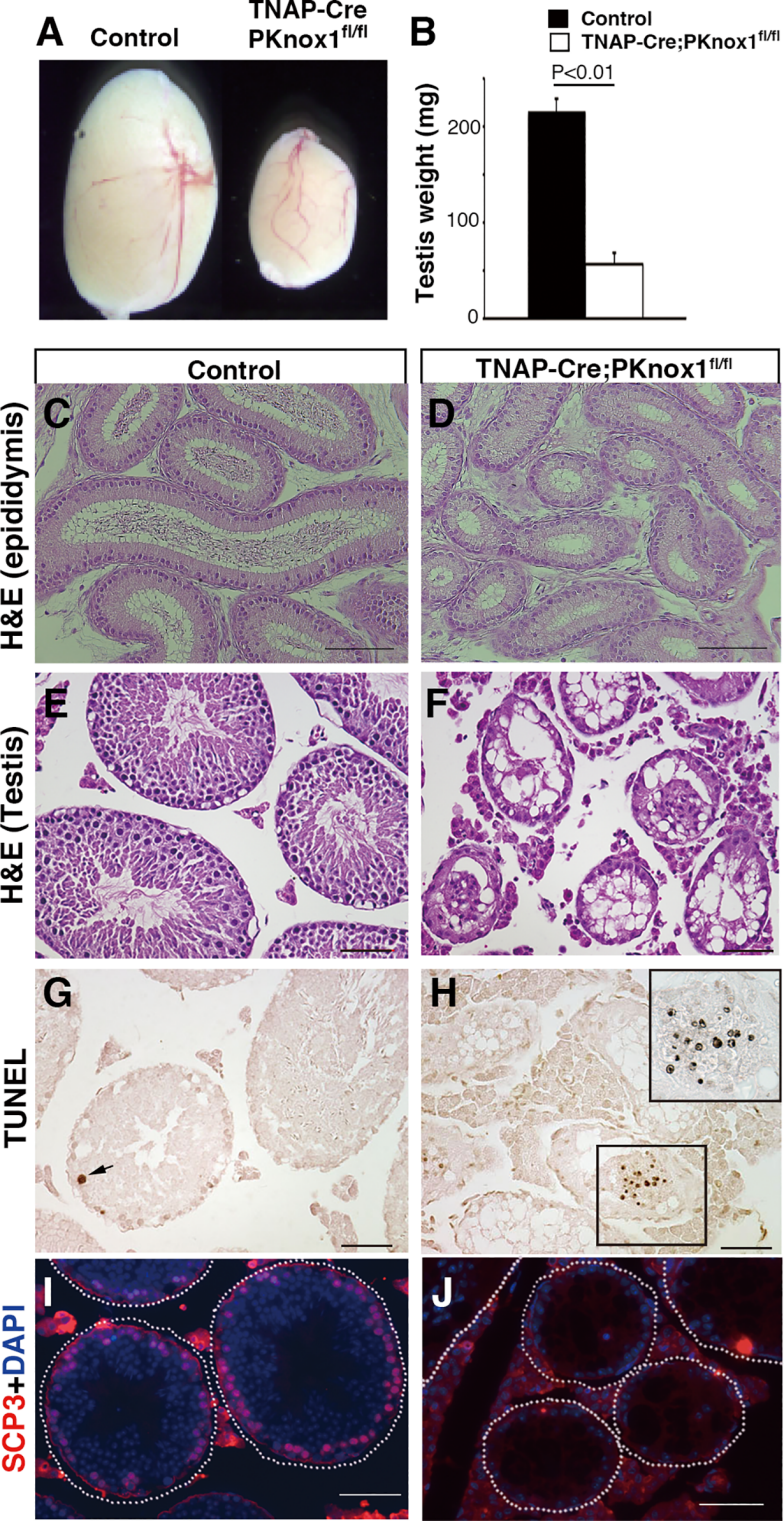
Germ cell-specific PKnox1 loss causes defective spermatogenesis. The size (**A**) and weight (**B**) of testes from 12-week-old PKnox1-GKO and control mice. Bars are the mean and standard deviation of the weight of testes. (n = 4 for each genotype). Tissue sections from control and GKO epididymis stained with H&E (**C**, **D**) and testes stained with H&E (**E, F**), TUNEL (**G, H**) and anti-SCP3 antibody with DAPI (**I, J**). Data are representative of 4 independent experiments. Scale bars, 50 μm.

While the preceding data suggested that *PKnox1* deficiency caused the differentiation arrest in spermatogenesis in the adult testes, *Rosa26-CreER*^*T2*^-mediated *PKnox1* deletion is not limited to germ cells. Thus, to determine whether the PKnox1-deficient spermatogenesis phenotype reflects germ cell-intrinsic or -extrinsic effects of *PKnox1* deletion, we crossed *PKnox1*^*fl*^ mice with *TNAP-Cre* mice[29], with germ cell-specific Cre activity, to generate germ cell-specific PKnox1-deficient mice (*TNAP-Cre; PKnox1*^*fl/fl*^: referred to as GKO) and *TNAP-Cre; PKnox1*^*fl/+*^ control mice. The phenotype of the testes in GKO mice was similar but even more severe than that observed in the PKnox1-CKO mice, which is probably due to the residual PKnox1 activity in germ cells from CKO mice that retained the remaining floxed *PKnox1* allele in the testes (S1B and S1C Fig). The size and weight of the testes from PKnox1-GKO mice (3-month-old) were significantly lower than those from littermate controls (Fig 3A and 3B). The epididymides from PKnox1-CKO mice lacked sperm (Fig 3C and 3D), and almost all the seminiferous tubules were atrophied with a loss of germ cells in the PKnox1-GKO testes (Fig 3E and 3F). An accumulation of TUNEL^+^ apoptotic cells inside the lumen was observed in a few tubules, probably the remaining apoptotic germ cells before their clearance from the tissue (Fig 3G and 3H). Furthermore, the PKnox1-GKO testes contained no SCP3^+^ meiotic cells (Fig 3I and 3J). Taken together, these findings indicate a germ cell-intrinsic requirement for PKnox1 in adult spermatogenesis.

### Defects in the spermatogonia populations upon PKnox1 loss

**Fig 4:**
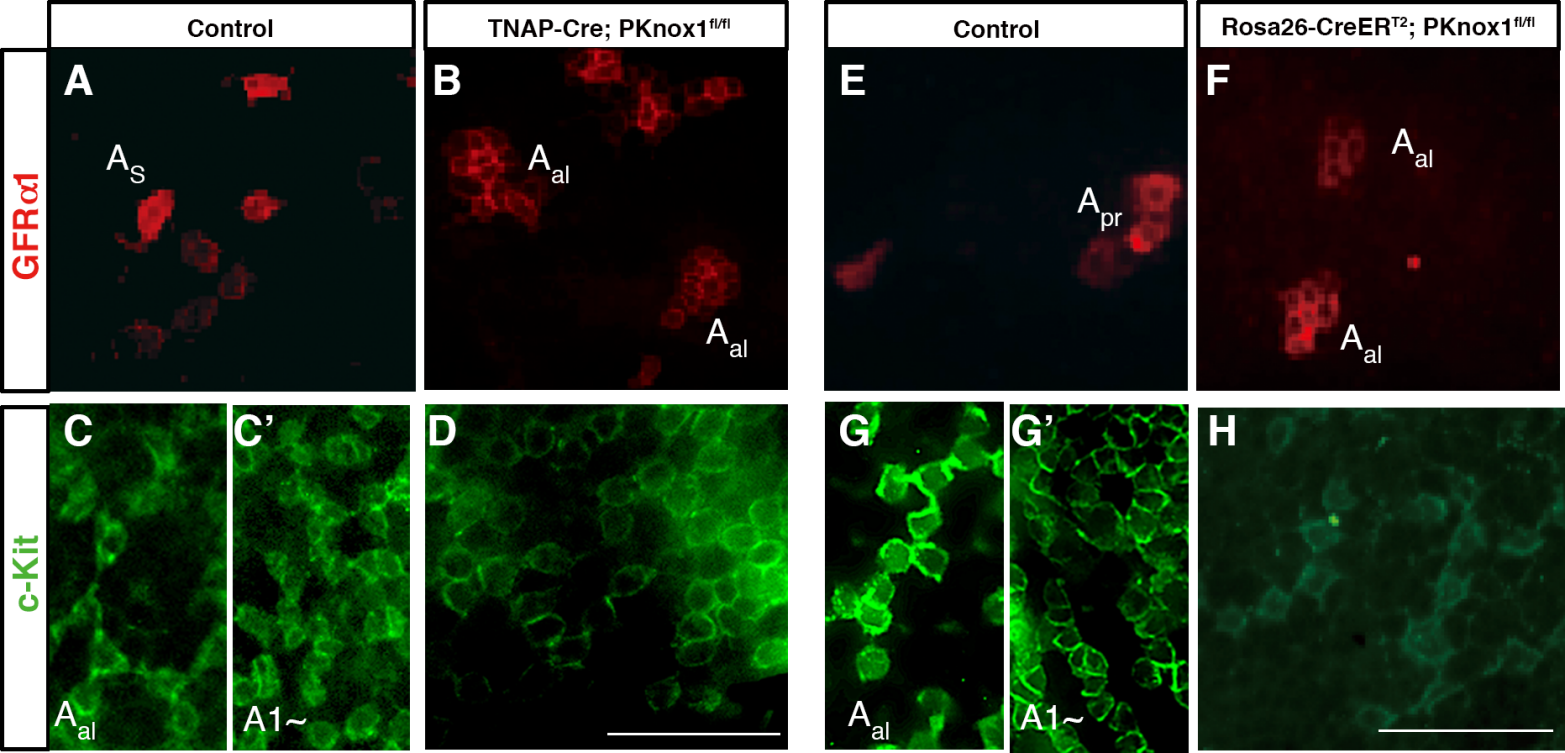
Loss of PKnox1 causes accumulation of GFRα1^+^ cells and differentiation arrest of c-Kit^+^ spermatogonia. Whole-mount immunodetection of cells expressing GFRα1 (red) and c-Kit (green) in seminiferous tubules of 12-week-old littermate controls (**A, C, C’, E, G and G’**), PKnox1-GKO (**B, D**), and -CKO mice (**F, H**). A_s_; single cell, A_pr_; paired cells, A_al_; aligned cells. Data are representative of 3 independent experiments. Scale bars, 50μm.

We further examined the differentiation of spermatogonia in the PKnox1-GKO and–CKO testis by whole-mount immunohistochemistry. This analysis reveled that GFRα1^+^ cells, corresponding to A_s~pr_ spermatogonia in the control testis (Fig 4A and 4E), form A_al_-like morphologies in both PKnox1-GKO and -CKO testes (Fig 4B and 4F). Furthermore, various developmental stages of c-Kit^+^ cells, including late undifferentiated (A_al16-32_) and differentiating spermatogonia (A1~), were observed in distinct areas of the seminiferous tubules from control testes, while the PKnox1-GKO testes contained relatively few c-Kit^+^ cells in which the level of c-Kit expression was weak, as compared to the control testes (S2 Fig). The detailed analysis revealed that c-Kit^+^ cells aligned more than 32 appear to be absent in the PKnox1-GKO and -CKO testes (Fig 4D and 4H), in contrast to the presence of A_al_ and A1-type spermatogonia in the control testes (Fig 4C, 4C’, 4G and 4G’).

**Fig 5:**
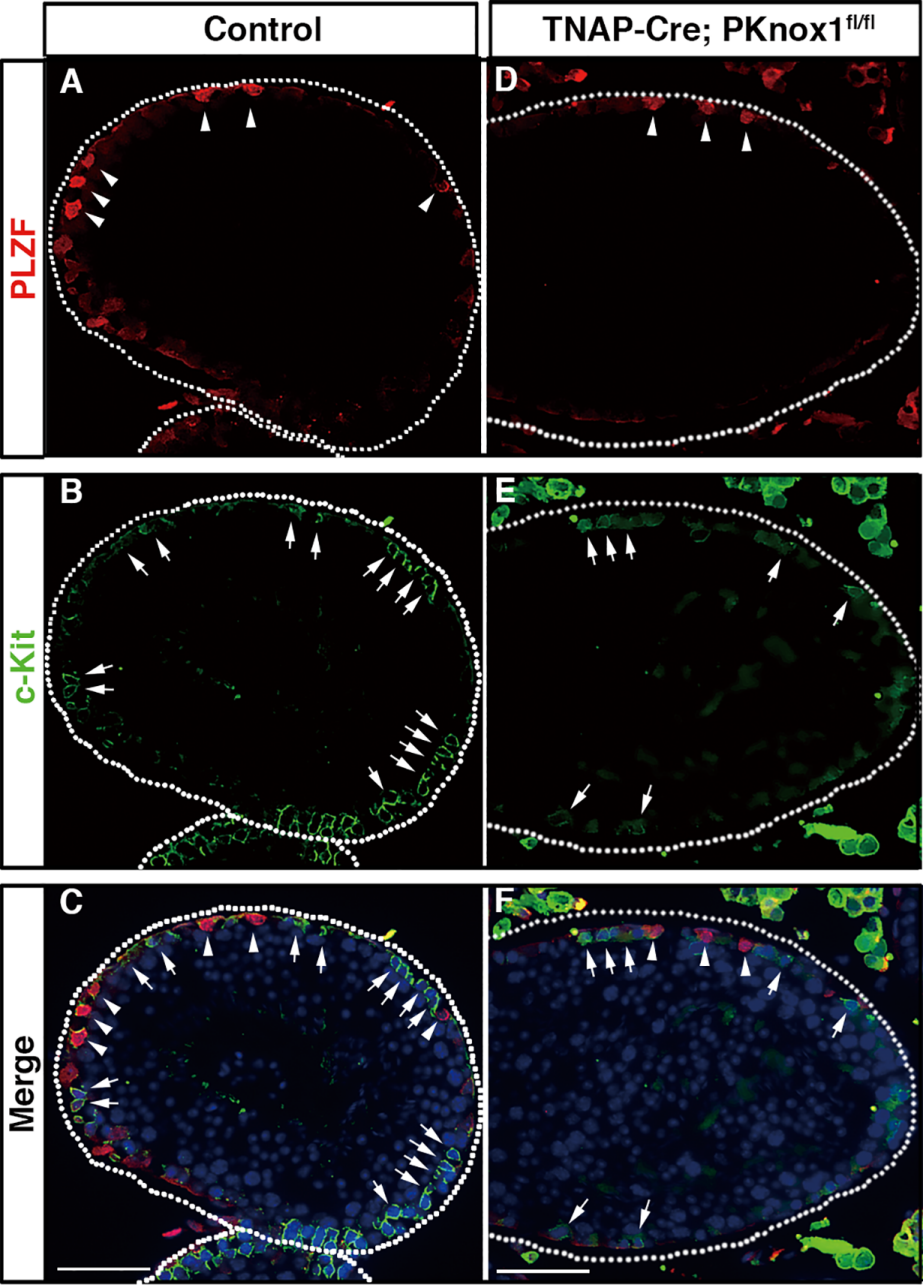
PLZF and c-Kit are expressed in a distinct subset of spermatogonia in PKnox1-deficient testes. Immunohistochemical analysis of PLZF (**A, D**) and c-Kit (**B, E**) expression in the testis from 12-week-old PKnox1-GKO and control mice with merged images (**C**, **F**). Tissue sections were double stained with the indicated combinations of antibodies and counterstained with DAPI. Arrowheads and arrows indicate PLZF^+^ and c-Kit^+^ cells, respectively. Broken lines indicate the basement membrane of seminiferous tubules. Data are representative of 3 independent experiments. Scale bars, 50 μm.

To further understand the defects in spermatogonia differentiation in the PKnox1-deficient testes, we examined whether sequential differentiation of spermatogonia occurs in PKnox1-GKO testes by simultaneous identification of markers differentially expressed during early to late spermatogonia differentiation, PLZF and c-Kit. PLZF is a transcription factor expressed in the early stage of spermatogonia differentiation, and is required not only for self-renewal[6,7] but also for the maintenance of spermatogonial stem cells in an undifferentiated state by repressing transcription of the *c-Kit* gene[30]. Thus, the expression of c-Kit begins after the cessation of PLZF expression at the later stage of spermatogonial cell differentiation. As similar to the non-overlapping pattern of PLZF and c-Kit expression in the control testes (Fig 5A–5C), distinct populations of germ cells expressed PLZF and c-Kit in the PKnox1-GKO testes (Fig 5D–5F), suggesting that PKnox1 is dispensable for initial differentiation of undifferentiated spermatogonia to the c-Kit^+^ stage.

### PKnox1 is required for differentiation of the c-Kit^+^ spermatogonia

**Fig 6:**
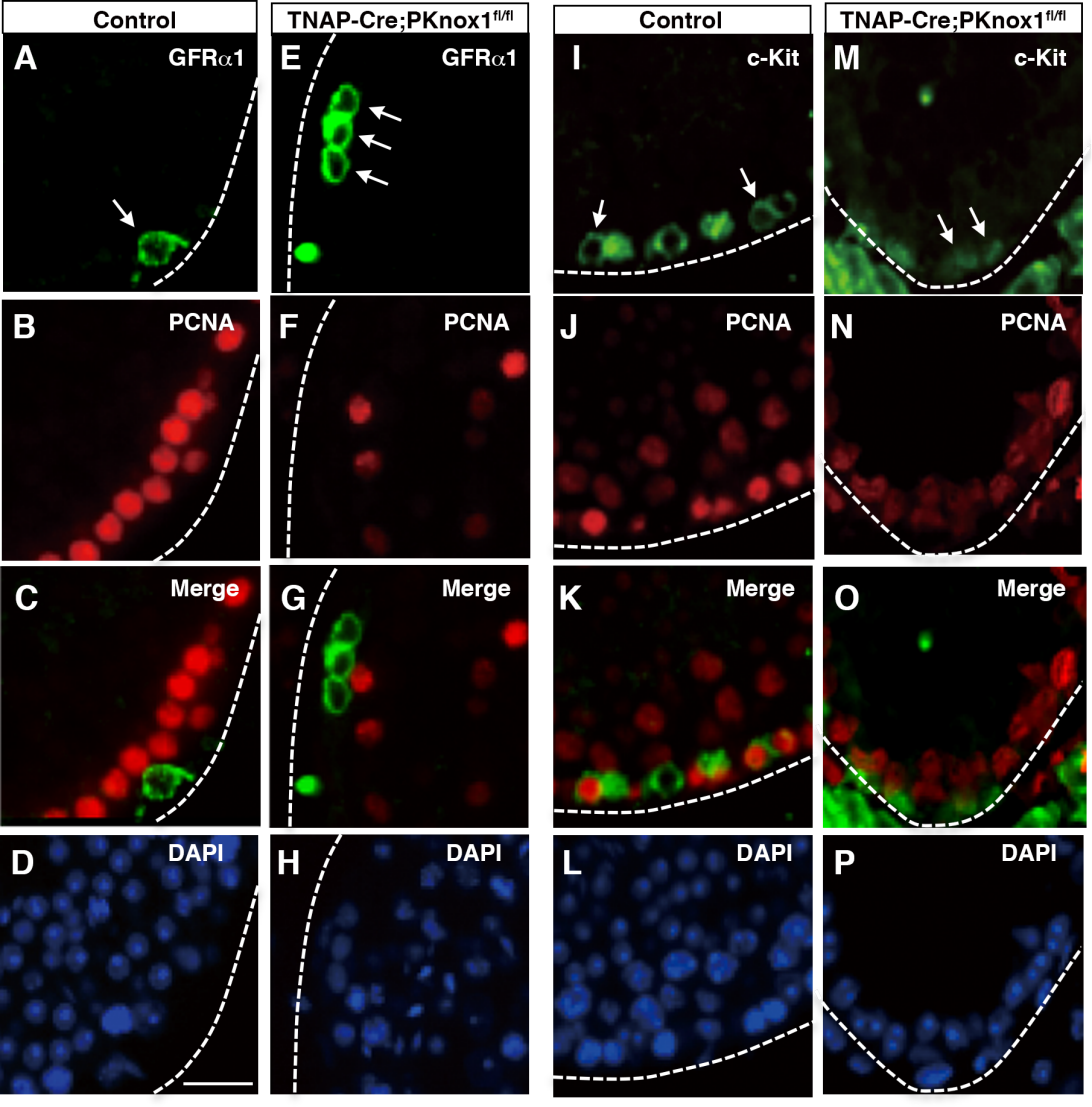
Loss of PKnox1 affects PCNA expression in c-Kit^+^ spermatogonia. Tissue sections from the testis from 12-week-old PKnox1-GKO and control mice were double stained with the indicated antibody combinations and counterstained with DAPI. Arrows indicate GFRα1^+^ and c-Kit^+^ cells with or without PCNA expression. Broken lines indicate the basement membrane of seminiferous tubules. Data are representative of 3 independent experiments. Scale bars, 25 μm.

Given that spermatogenesis proceeded at least to the c-Kit^+^ spermatogonial differentiation stage but subsequent stages including meiosis were absent in the PKnox1-GKO testes, we finally focused our attention on alterations in the c-Kit^+^ stage of spermatogonia differentiation, when rapid cell proliferation occurs through signals from c-Kit[31]. In the control testes, GFRα1^+^ cells, representing the most immature spermatogonial cells, were negative for PCNA, one of markers of proliferating cells (Fig 6A–6D), whereas c-Kit^+^ spermatogonial cells expressed a high level of PCNA(Fig 6I–6L). Although GFRα1^+^ cells in in the PKnox1-GKO testes were PCNA-negative (Fig 6E–6H), as similar to the control testes, c-Kit^+^ spermatogonia in the PKnox1-GKO testes appeared to be negative for PCNA (Fig 6M–6P**)**, suggestive of a possibility that proliferation defects in PKnox1-defficient c-Kit^+^ spermatogonia might be associated with the subsequent differentiation arrest of spermatogenesis.

## Discussion

Here we demonstrate that PKnox1 is critical for the maintenance of spermatogenesis in the post pubertal testis and is prerequisite for sustained spermatogenesis in adult male mice. Tamoxifen-mediated deletion of *PKnox1* in the adult testes as well as its germ cell-selective ablation causes testis hypotrophy with germ cell apoptosis and, as a consequence, compromised spermatogenesis resulting in the loss of germ cells at the stage of meiosis.

Upon PKnox1 inactivation, the undifferentiated spermatogonia expressing GFRα1 appeared to form clusters. There might be two potential explanations for this phenotype after PKnox1 loss, abnormal differentiation and/or proliferation of spermatogonia. In this regard, while c-Kit^+^, but not GFRα1^+^, spermatogonia in the control testes expressed the cell proliferation marker, PCNA, neither of these spermatogonia populations in PKnox1-GKO testes expressed PCNA. Thus, the appearance of A_al_-like GFRα1^+^ spermatogonia is not likely to be a consequence of proliferation of GFRα1^+^ spermatogonia themselves. Furthermore, as PLZF represses c-Kit transcription in the A_s_ and A_pr_ undifferentiated spermatogonia to maintain these cells in the G1/G0 phase of cell cycle[30], PLZF expression was not detected in the more differentiated c-Kit^+^ stage of spermatogonia in the PKnox1-GKO testes, indicating that differentiation of GFRα1^+^spermatogonia to the c-Kit^+^ stage occurs in the absence of PKnox1. Therefore, it seems reasonable to propose that the accumulation of undifferentiated GFRα1^+^ spermatogonia observed in the absence of PKnox1 results from an indirect consequence of a failure in maintenance and/or subsequent differentiation of the more differentiated c-Kit^+^ stage of spermatogonia[32]. Combining the previous findings that spermatogonia enter the S phase of cell cycle after differentiation into the A1 stage[33,34] and our present data that PKnox1-deficient c-Kit^+^ spermatogonia were PCNA-negative, the prime spermatogenic defect in PKnox1 deficiency appears to be a failure of the c-Kit^+^ A_al_ spermatogonia to differentiate into A1 spermatogonia. As a result, most seminiferous tubules of PKnox1-deficient testes only contain A_s_, A_pr_ and A_al_ spermatogonia, with a loss of subsequent differentiation stages of germ cells.

The importance of c-Kit-mediated signaling, particularly that mediated by phosphatidylinositol 3’-kinase (PI3K), in spermatogenesis is well established[12]. Mice bearing a point mutation in c-Kit that specifically disrupts a binding site for the p85 subunit of PI3-kinase fail to produce sperm due to decreased proliferation by the cKit^+^ spermatogonia and increased apoptosis, probably caused by the arrest of subsequent spermatogenic differentiation[35]. In the PKnox1-deficient testes, A_al_ spermatogonia expressed c-Kit, albeit its expression level was weaker than that of controls, and appeared to fail to proliferate, suggesting possible defects in c-Kit-mediated signaling. Although there have been no studies indicating a possible function of PKnox1 in c-Kit-mediated signaling, Meis3, another member of the PKnox/Meis family of transcription factors, was reported to transcriptionally regulate expression of PDK1, a serine/threonine kinase functioning downstream of PI3K[36]. The potential involvement of PKnox1 in c-Kit-mediated signaling warrants further investigation.

Considering the binding partners and potential upstream regulators of PKnox1 expressed in the spermatogenic process, Pbx4 is preferentially expressed in pachytene spermatocytes corresponding to meiotic spermatocytes, but not in spermatogonia[23]. In addition, a potential upstream regulator of TALE family transcription factors, MLL5, is thought to participate only in the late stage of spermatogenesis, as its deficiency causes defects predominantly in the post meiotic stages of spermatogenesis that lead to spermatozoa[37]. Since PKnox1 is expressed not only in spermatogonia but also in haploid germ cells, PKnox1 might have additional roles in the post meiotic stage of spermatogenesis in conjunction with Pbx4 and MLL5, such as activation of *Pgk2* gene expression, as previously suggested[24,25].

Overall, our findings strongly indicate that PKnox1 represents a new intrinsic regulator of maintenance and subsequent differentiation of the c-Kit^+^ stage of spermatogonia, adding important insight into our understanding of the molecular mechanisms regulating spermatogenesis, a process that is essential for sustaining the germline in adulthood and is therefore required for male fertility.

## Materials and methods

### Ethics statement

All animal experiments were carried out under the ethical guidance of Tokyo University of Science, and protocols were reviewed and approved by the Tokyo University of Science Animal Care and Use Committee.

### Mice and gene targeting

All animal experiments were carried out in accordance with the ethical guidance of Tokyo University of Science, and protocols were reviewed and approved by the Tokyo University of Science Animal Care and Use Committee. All of the genomic fragments used in constructing the targeting vector for the *PKnox1* conditional allele, including a 0.9-kb genomic fragment immediately upstream of the *lox*P-flanked 1.2-kb fragment containing exon 3 of the *PKnox1* gene and a 5.5-kb fragment immediately downstream of exon 3, were obtained from a C57BL/6 bacterial artificial chromosome (BAC) clone (RP23-99E16, Invitrogen) by using the RED/ET system (Gene Bridge GmbH, Heidelberg, Germany). These fragments were assembled in a modified pBluescript II SK vector containing a PGK promoter-driven FRT-flanked neomycin-resistant gene and an MC1 promoter-driven diphtheria toxin gene. The linearized targeting vector was electroporated into Bruce-4 ES cells and drug-resistant colonies were screened for homologous recombination. Targeted clones were injected into BALB/c blastocysts and the resultant chimeric mice were bred to produce progeny with germ line transmission of the mutated allele. F1 progeny harboring a targeted *PKnox1* allele were then crossed with ubiquitous *CAG* promoter-driven FLPe mice on a C57BL/6 background[38] (obtained from RIKEN Bioresource Center) to remove the FRT-flanked neomycin-resistant gene cassette. *Rosa26-CreER*^*T2*^[28] and *TNAP-Cre* mice[29] on a C57BL/6 background were provided from Drs. T. Ludwig and A. Nagy, respectively. C57BL/6, BALB/c, and *W/W*_v_ mice were purchased from Japan SLC Inc. (Hamamatsu Japan). Mice were euthanized under general anesthesia with an isoflurane overdose (5%). The PCR primers for genotyping are listed in **S1 Table**.

### Tamoxifen treatment

Tamoxifen (final concentration: 10 mg/ml, Sigma-Aldrich, St Louis, MO) was prepared as described previously[39]. In brief, 50 mg of tamoxifen was dissolved into 500 μl of ethanol at 55°C followed by addition of 4.5 ml of warmed sunflower oil and mixed thoroughly by vortexing. The solution was then filtered and stored at -20°C. For *in vivo* administration, 5 mg tamoxifen/30 g per body weight was delivered by intragastric gavage for three consecutive days to 3-month-old male mice.

### RT-PCR

Total RNA was isolated using Trizol reagent (Invitrogen) and first-strand cDNA was synthesized using oligo (dT) primers and the Superscript RT-PCR kit (Invitrogen). Each cDNA sample was then subjected to PCR. Amplified signals were confirmed to be single bands by gel electrophoresis and were normalized to β-actin. Primer sequences and PCR conditions are listed in **S1 Table**.

### Immunoblotting

Testes lysed in RIPA buffer were separated by SDS-PAGE and transferred to PVDF membranes. The blots were then probed with anti-PKnox1/Prep1 (Abcam, Cambridge, MA, USA) and anti-GAPDH (Santa Cruz, CA) antibody, followed by HRP-conjugated secondary antibodies and then visualized by enhanced chemiluminescence (ECL plus). Blots were scanned and analyzed using a Luminescent image analyzer (LAS-3000, FUJIFILM, Japan).

### Immunohistochemistry

The testis samples were fixed with 4% paraformaldehyde (PFA) in PBS at 4°C for 16 hours, dehydrated in ethanol, cleared in xylene, and then routinely embedded in paraffin. The deparaffinized tissue sections were blocked with PBS containing 3% bovine serum albumin (Sigma) then incubated with primary antibodies diluted in Can Get Signal Immunostain buffer (Toyobo) at 4°C for 12 hours followed by a one-hour incubation at room temperature with the appropriate secondary antibodies. The following primary antibodies were used: rabbit anti-SCP3 (1:100 dilution; Santa Cruz), goat anti-mouse c-Kit (1:100 dilution; R&D systems), goat anti-mouse GFRα1 (1:100 dilution; R&D systems), mouse anti-PCNA (1:100 dilution; Santa Cruz), mouse anti-PLZF (1:100 dilution; Santa Cruz) and mouse anti-PKnox1/Prep1 (1:100 dilution; Abcam). Secondary antibodies were anti-rabbit IgG antibody conjugated with Alexa Fluor 488 or 647 (Cell Signaling), anti-mouse IgG antibody conjugated with Alexa Fluor 633 or 488 (Invitrogen), or anti-goat IgG antibody conjugated with Alexa Fluor 633. Nuclei were counterstained with Prolong Gold antifade reagent with DAPI (Invitrogen). For whole-mount immunohistochemistry, testes were removed from the tunica albuginea, fixed in 4% PFA for 16 hours at 4°C and washed with cold PBS. The seminiferous tubule fragments were incubated with goat anti-mouse GFRα1 (1:100 dilution; R&D systems), goat anti-mouse c-Kit (1:100 dilution; R&D systems) antibodies at 4°C for 12 hours. After washing with PBS, the samples were incubated with Alexa-488/555 conjugated secondary antibodies at room temperature for two hours. After counter-staining with DAPI, the samples were analyzed using a BIOREVO BZ-9000 microscope (KEYENCE). Whole mount samples of the seminiferous tubule were photographed (×400).

### Apoptosis analysis

Paraffin sections were deparaffinized and subjected to the terminal deoxynucleotydyl transferase-mediated dUTP nick-end labeling (TUNEL) assay using the *In Situ* Cell Death Detection Kit (Roche, USA). The TUNEL working procedure was carried out following the manufacturer's protocols.

### Statistical analysis

Statistical significance was calculated with the unpaired two-tailed Student’s *t*-test. Data were considered statistically significant when p values were less than 0.05.

## Supporting information

S1 Fig Generation of the conditional *PKnox1* allele. (**A**) Diagram depicting exon 3 of the *Prep1* locus and the targeting strategy used to generate mutant *PKnox1* alleles (floxed and deleted alleles). *LoxP* sites (arrowheads) were inserted into intronic sites flanking exon 3 of the *PKnox1* gene. The FRT-flanked neomycin gene (PGK-neo) selection cassette was removed by crossing to CAG-FLPe mice. PCR primers for verifying the Cre-mediated deletion of the lox*P*-flanked fragment are indicated by arrows. DT-A, a diphtheria toxin negative selection cassette; B, Bam*HI*: E, *Eco*RI. (**B**) Confirmation of *PKnox1* deletion. Genomic DNAs from testes of *Rosa26-CreER*^*T2*^*; PKnox1*^*fl/fl*^ and *Rosa26-CreER*^*T2*^*; PKnox1*^*fl/+*^ mice that were treated with tamoxifen 3 weeks previously were subjected to PCR analysis using the indicated primer pairs. (**C**) Western blot analysis to confirm loss of PKnox1 protein expression. Whole-cell lysates from testes of *Rosa26-CreER*^*T2*^*; PKnox1*^*fl/fl*^ and *Rosa26-CreER*^*T2*^*; PKnox1*^*fl/+*^ mice that were treated with tamoxifen 3 weeks previously were blotted with an anti-PKnox1 antibody. After stripping, the filter was reprobed with an anti-GAPDH antibody.
(TIF)

S2 Fig Whole-mount immunohistochemistry of c-Kit^+^ cells in PKnox1-GKO testes. Distribution of c-Kit^+^ cells in the seminiferous tubules of 12-week-old littermate controls (upper panels) and PKnox1-GKO mice (lower panels). Arrows indicate c-Kit^+^ cells in PKnox1-GKO testes. Asterisks indicate the area of the seminiferous tubules lacking c-Kit^+^ cells in PKnox1-GKO testes.
(TIF)

S1 Table List of primers for genotyping and RT-PCR. (DOC)
