# Supplementary figures and images for: Germ cell-intrinsic requirement for the homeodomain transcription factor PKnox1/Prep1 in adult spermatogenesis
Source: PLoS One. 2018 Jan 2;13(1):e0190702. doi: 10.1371/journal.pone.0190702 (PMC5749842; doi:10.1371/journal.pone.0190702)

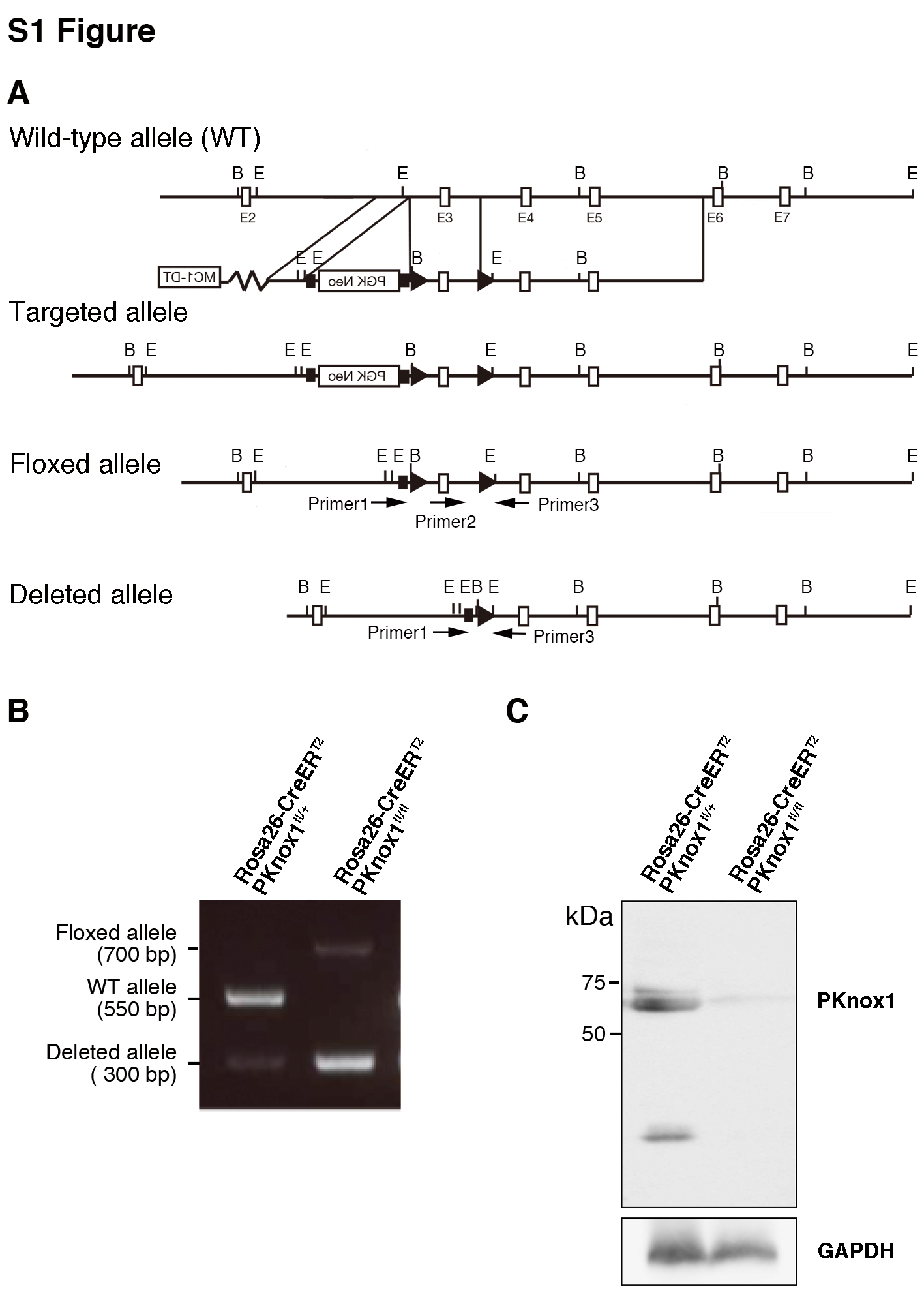

Supplement: S1 Fig — (A) Diagram depicting exon 3 of the Prep1 locus and the targeting strategy used to generate mutant PKnox1 alleles (floxed and deleted alleles). LoxP sites (arrowheads) were inserted into intronic sites flanking exon 3 of the PKnox1 gene. The FRT-flanked neomycin gene (PGK-neo) selection cassette was removed by crossing to CAG-FLPe mice. PCR primers for verifying the Cre-mediated deletion of the loxP-flanked fragment are indicated by arrows. DT-A, a diphtheria toxin negative selection cassette; B, BamHI: E, EcoRI. (B) Confirmation of PKnox1 deletion. Genomic DNAs from testes of Rosa26-CreERT2; PKnox1fl/fl and Rosa26-CreERT2; PKnox1fl/+ mice that were treated with tamoxifen 3 weeks previously were subjected to PCR analysis using the indicated primer pairs. (C) Western blot analysis to confirm loss of PKnox1 protein expression. Whole-cell lysates from testes of Rosa26-CreERT2; PKnox1fl/fl and Rosa26-CreERT2; PKnox1fl/+ mice that were treated with tamoxifen 3 weeks previously were blotted with an anti-PKnox1 antibody. After stripping, the filter was reprobed with an anti-GAPDH antibody. (TIF) [file pone.0190702.s001.tif]

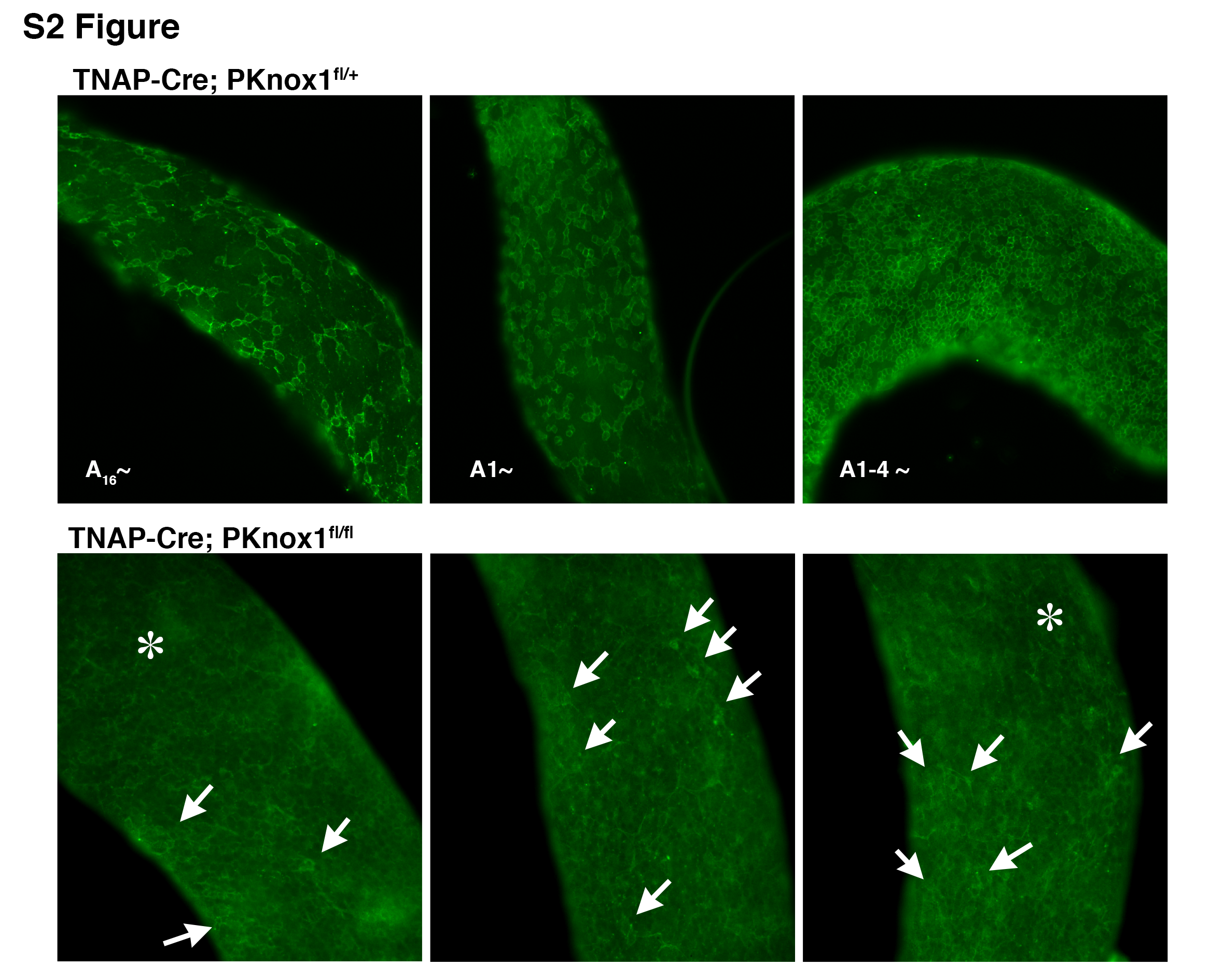

Supplement: S2 Fig — Distribution of c-Kit+ cells in the seminiferous tubules of 12-week-old littermate controls (upper panels) and PKnox1-GKO mice (lower panels). Arrows indicate c-Kit+ cells in PKnox1-GKO testes. Asterisks indicate the area of the seminiferous tubules lacking c-Kit+ cells in PKnox1-GKO testes. (TIF) [file pone.0190702.s002.tif]
