# Supplementary material for: Germ cell-intrinsic requirement for the homeodomain transcription factor PKnox1/Prep1 in adult spermatogenesis
Source: PLoS One. 2018 Jan 2;13(1):e0190702. doi: 10.1371/journal.pone.0190702 (PMC5749842; doi:10.1371/journal.pone.0190702)
Supplement: S1 Table — (DOC) [file pone.0190702.s003.doc]

| **S1 Table. Primer sequences used for genotyping and RT-PCR.** | | |  |
| --- | --- | --- | --- |
|  |  |  |  |
| allele name |  | Primer sequence |  |
| **Genotyping** |  |  |  |
| Primer1 | F: | GGATCCTAGTGAACCTCTTCGAGG |  |
| Primer2 | F: | GACCTTCACATAGCTGCGCAGTTG |  |
| Primer3 | R: | CGCACAGGAAGATGAGTGACTGC |  |
|  |  |  |  |
| Rosa26-CreER^T2^ | F: | CTGGGAGTTCTCTGCTGCCTCCTGG |  |
|  | R: | GCATAACCAGTGAAACAGCAT |  |
|  |  |  |  |
| TNAP-Cre | F: | CCAGAAGGTACCCCATTGTATGG |  |
|  | R: | GCATAACCAGTGAAACAGCAT |  |
|  |  |  |  |
| **RT-PCR** |  |  |  |
| PKnox1 | F: | AGCAGGCCATTTATAGGCATC |  |
|  | R: | TCACCATTAGGTTGTCAGTTTCC |  |
|  |  |  |  |
| β-actin | F: | CTACAATGAGCTGCGTGTGG |  |
|  | R: | AAGGAAGGCTGGAAGAGTGC |  |
|  |  |  |  |
| F; Forward, R;Reverse |  |  |  |
